# AI testing, evaluation, verification and validation for accessibility: a comprehensive framework

**DOI:** 10.3389/fdgth.2025.1679603

**Published:** 2026-02-26

**Authors:** Gabriella Waters

**Affiliations:** 1 Cognitive and Neurodiversity AI Lab, Center for Responsible AI, Virginia State University, Baltimore, VA, United States; 2 Center for Equitable AI and Machine Learning Systems, Morgan State University, Baltimore, VA, United States

**Keywords:** accessibility (for disabled), AI evaluation, AI evaluation framework, AI testing, artificial intelligence

## Abstract

As artificial intelligence (AI) systems continue to remain prevalent in society, ensuring their accessibility for all users, including those with disabilities, is of great importance. This paper presents a comprehensive framework for AI Testing, Evaluation, Verification and Validation (TEVV) focused on accessibility. The proposed methodology incorporates methods for red teaming, model testing, and field testing with a particular emphasis on usability testing for accessibility. The results demonstrate, through detailed case studies, that systematically evaluating AI systems for accessibility barriers and biases improves the inclusivity and effectiveness of AI technologies for diverse user populations. The findings suggest that this accessibility-focused TEVV framework provides a structured approach for developing more equitable and universally usable AI systems that benefit all members of society.

## 1 Introduction

Artificial intelligence (AI) technologies are rapidly transforming numerous aspects of modern life, from healthcare and education to transportation and communication. As these systems continue to become more deeply integrated into essential services and daily interactions, making sure they are accessible for all users, including those with disabilities, is not just a matter of inclusivity but a fundamental necessity.

Accessibility in AI refers to the design and development of AI systems that can be effectively used by people with a wide range of abilities and disabilities. This includes considerations for visual, auditory, motor, and cognitive impairments, as well as neurodiversity and situational limitations. Prioritizing accessibility in AI development and deployment, allows for the creation of more equitable technologies that empower all individuals to participate fully in an increasingly AI-driven world (1).

However, achieving true accessibility in AI systems presents unique challenges. Unlike traditional software applications, AI models often exhibit complex, dynamic behaviors that can be difficult to predict and evaluate comprehensively (2). Additionally, harmful biases in training data and algorithmic design can lead to discriminatory outcomes that disproportionately affect users with disabilities.

Despite progress in digital accessibility, most AI systems are evaluated using practices that prioritize accuracy and generalizability over inclusivity, which can lead to persistent access barriers for individuals with disabilities. The TEVV framework reconceptualizes evaluation as an iterative, multi-layered process that centers accessibility from project inception through deployment, bridging the gap between regulatory compliance, technical rigor, and user experience with diverse populations.

This is where a robust framework for AI testing, evaluation, verification and validation (TEVV) focused on accessibility becomes necessary. Systematic assessment of AI systems for potential barriers, harmful biases, and usability issues related to accessibility, can help to identify and address problems early in the development process. This proactive approach not only improves the inclusivity of AI technologies but also enhances their overall effectiveness and user satisfaction across diverse populations.

The TEVV for accessibility framework was developed through an integrative literature synthesis and conceptual modeling approach. Key components were derived from a review of accessibility standards (ISO 9241-210; WCAG 2.1), empirical and meta-analytic findings in AI accessibility and evaluation (1, 2), and best practices identified in human-computer interaction and disability studies. Iterative comparison with published frameworks enabled the refinement of unique metrics, taxonomy structures, and process stages. Expert commentaries and consensus reports from the literature (3, 4) guided the framework's multi-modal and continuous validation orientation. No original human or empirical data collection was conducted; all recommendations, examples, and use-case scenarios are derived or adapted from prior research syntheses and are illustrative of how the framework can be operationalized in real-world contexts (5, 6).

The proposed framework builds upon existing TEVV methodologies while introducing specialized techniques for evaluating accessibility in AI contexts. It incorporates methods for red teaming, which simulates adversarial scenarios to uncover potential accessibility vulnerabilities; model testing to rigorously evaluate AI model performance across diverse user profiles; field testing to assess real-world utility and accessibility in authentic contexts; and usability testing to conduct in-depth evaluations with users who have various disabilities. The framework aims to provide a comprehensive strategy for ensuring that AI systems are truly accessible and inclusive from the ground up through the integration of these approaches.

This paper will explore the key components of this accessibility focused TEVV framework throughout, compare it to current practices, and demonstrate its application through detailed example case studies. Adoption of this systematic approach to accessibility testing in AI development can contribute to the creation of more equitable, effective, and universally usable AI technologies that benefit all members of society.

### 1.2 Significance and contributions of this work

This paper attempts to address a critical gap in the field of AI development and evaluation by proposing a framework for testing, evaluation, verification, and validation specifically focused on accessibility. The rapid proliferation of AI systems across many domains of daily life in tandem with the potential of these technologies to exacerbate or alleviate barriers faced by individuals highlight the need for this work.

Current research in AI accessibility has primarily focused on specific applications or types of disabilities that frequently treat accessibility as an add on instead of a fundamental aspect of AI development. This paper contributes to the field by offering a holistic approach that integrates accessibility considerations throughout the entire AI lifecycle. The combination of methods from red teaming, model testing, field testing, and usability testing affords the proposed framework with a structured methodology for identifying and addressing a wide range of accessibility issues that may arise in AI systems.

While there has been significant work in AI ethics and fairness, there is a noticeable lack of standardized methodologies for comprehensive AI TEVV in the context of accessibility. The framework presented in this paper offers a systematic approach that can be adapted across different AI applications and domains. The framework's emphasis on iterative testing and diverse user involvement addresses the dynamic nature of AI systems and the varied needs of users with disabilities, which are aspects that are often overlooked in traditional AI testing methods.

The paper also makes contributions in the form of specific metrics and evaluation criteria for AI accessibility. These quantifiable measures provide developers and researchers with concrete tools to assess and improve the accessibility of their AI systems to facilitate more objective comparisons across the industry. The example case studies presented demonstrate the practical application and effectiveness of the proposed framework and offer insights into how organizations can implement comprehensive accessibility testing in real-world AI development scenarios. These approaches offer ways to bridge the gap between theoretical approaches and practical implementation.

By addressing the intersectionality of accessibility with other aspects of AI development like harmful bias mitigation and ethical considerations, this work can also contribute to a more nuanced understanding of inclusive AI design. Accessibility testing can uncover and address the issues that benefit all users, not just those with disabilities, making a case for accessibility as a driver of overall AI quality and usability.

Throughout the manuscript, we use several terms with closely related but distinct meanings as outlined in the Glossary of Key Terms below. Consistency in terminology enhances both the internal clarity of the framework and its alignment with ongoing scholarly and normative discourse.

Glossary of key terms

Accessibility: The design of AI systems to be usable by people with a range of abilities and disabilities, including sensory, motor, cognitive, and situational limitations (ISO 9241-210; WCAG 2.2).
Accessibility Gap: The measurable disparity between the accessibility features, outcomes, or experiences of disabled and non-disabled users in a system (7).
Equity: The process of ensuring fair treatment, opportunities, and outcomes for all individuals, with recognition of specific needs or barriers faced by marginalized groups, including people with disabilities (8).
Fairness: The absence of bias, discrimination, or favoritism toward any group in AI systems, including ensuring equal opportunity and treatment for people with disabilities (9).
Harmful Bias: Systematic error or unfair outcomes produced by an AI system due to data, design, or model artifacts that disadvantage certain groups (2).

## 2 Literature review

Recent research underscores the inadequacy of conventional, siloed evaluation methods for capturing the lived realities and evolving needs of users with disabilities. Studies from theoretical, empirical, and standards perspectives converge on the need for continuous, multi-modal validation, affirming the TEVV paradigm as an essential evolution in accessibility theory and practice,

### 2.1 Accessibility testing in AI

Chemnad and Othman's (2) bibliometrics analysis and systematic review of 43 articles from 2018 to 2023 that focused on AI applications for digital accessibility reveals a disproportionate focus on visual impairments compared to motor and cognitive disabilities, which highlights gaps in addressing speech/hearing impairments and neurodiverse needs. Their classification framework informs testing protocol development through technical, ethical, and implementation categories, with machine learning algorithms demonstrating capability to recognize patterns and identify accessibility barriers in digital content through image recognition for alternative text generation and natural language processing for cognitive accessibility support. This work is essential for AI TEVV for accessibility research as it provides a comprehensive taxonomy of accessibility testing approaches and identifies systematic gaps in current testing methodologies that must be addressed in verification and validation frameworks.

Fuglerud et al. (10) developed and evaluated machine learning prototypes for automated WCAG compliance checks through implementing computer vision algorithms for image analysis and natural language processing for content evaluation. Their experimental results demonstrated high precision rates for color contrast violations and alternative text assessment but revealed concerning false positive rates that required human intervention. The study compared automated detection capabilities against manual expert evaluation and found that AI systems excelled at identifying technical violations but struggled with contextual accessibility judgments, such as determining whether decorative images require alternative text descriptions. This evaluation of AI-powered accessibility testing tools directly supports TEVV frameworks by establishing performance benchmarks and identifying validation requirements for automated accessibility assessment systems.

Guo et al. (3) conducted a multi-round Delphi study with 24 AI ethics experts from academia and industry to identify critical gaps in AI accessibility evaluation. Through three rounds of structured questionnaires and consensus-building exercises, the study revealed three fundamental deficiencies: lack of assistive technology interoperability standards, absence of longitudinal evaluation methods accounting for progressive disabilities, and inadequate adaptive performance metrics that adjust to individual user capabilities (76% agreement). The research established expert consensus on priority areas for AI accessibility research, including the need for standardized testing protocols that can evaluate AI systems across diverse assistive technologies and the development of dynamic assessment frameworks that account for changing user needs over time. This expert consensus methodology provides foundational requirements for TEVV frameworks by establishing evidence-based priorities for comprehensive accessibility validation protocols that must be incorporated into continuous testing systems.

Nwokoye et al. (11) present a systematic survey of Accessible, Explainable Artificial Intelligence (AXAI) that examines how explanations of AI decisions are made accessible that highlights similar gaps at the intersection of explainability and disability inclusion as this work. The authors focus on people with sight loss and other visual challenges and highlight that most explainable AI techniques continue to rely on visually oriented modalities (e.g., charts, graphical overlays) that often exclude blind and low-vision users unless non-visual or low-vision optimized channels are provided (e.g., haptic or auditory feedback). Their findings align with the present paper's argument that accessibility must be addressed across the full TEVV lifecycle. This includes core functionality, model transparency, interpretability, and user-facing explanations as integral parts of accessibility evaluation vs. optional add-ons.

### 2.2 Harmful bias in AI accessibility

El Morr et al. (8) conducted a systematic scoping review analyzing 64 peer-reviewed studies on AI and disability to examine bias manifestations across AI systems. Their analysis revealed three primary mechanisms of bias amplification: dataset underrepresentation, where training data contained insufficient representation of disabled users; ableist problem framing, where AI development prioritized normative abilities over accessibility needs; and inappropriate evaluation metrics, which failed to account for diverse interaction patterns. The review demonstrated that AI systems consistently performed worse for disabled users across multiple domains, with facial recognition showing higher error rates for wheelchair users and voice recognition exhibiting reduced accuracy for users with speech disabilities. This comprehensive bias analysis provides essential validation requirements for TEVV frameworks by establishing systematic approaches to identify and measure discriminatory outcomes in AI accessibility systems. It establishes verification requirements for harmful bias detection and validation protocols that should be considered for integration into testing frameworks to ensure equitable AI system performance across diverse disability populations.

Kane et al. (12) conducted an online survey with 40 adults with physical disabilities across the United States to gather open-ended descriptions about participants' experiences with various sensing systems including motion sensors, biometric sensors, speech input, and touch/gesture systems. Their qualitative analysis used affinity diagramming and identified ten key challenge areas that sensing technologies present for people with physical disabilities: premature timeouts, poor device positioning, being “invisible” to sensors, mismatches between users' abilities and sensors' fidelity for range of motion, variability of users' abilities over time, difficulty setting up sensing systems, biometric failures, security vulnerabilities, incorrect inferences, and data validation problems. The study also revealed four patterns of response that participants used to mitigate these challenges: seeking assistance from others, developing custom adaptations, avoiding sensing technologies entirely, and abandoning technologies altogether. Their findings demonstrated specific accessibility barriers including automatic doors timing out before wheelchair users could pass through, sensor buttons positioned too high or in awkward locations for wheelchair access, motion sensors failing to detect wheelchair users due to mounting height and angle issues, and step-counters generating invalid data when used by wheelchair users rather than pedestrians. This comprehensive mixed-methods evaluation methodology provides validation protocols for TEVV frameworks by demonstrating how to systematically document and categorize accessibility barriers in AI-powered sensing systems through structured user experience research that captures both quantitative prevalence data and qualitative insights into real-world usage challenges.

Trewin et al. (9) conducted a community-centered, design-oriented inquiry into fairness in AI systems as it relates to people with disabilities. The authors presented empirical case studies and analyses drawn from industry, research, and accessibility advocacy. The authors examined multiple real-world AI systems, such as automated hiring tools, facial recognition, and virtual assistants, to illustrate how standard definitions of fairness often overlook or disadvantage users with disabilities. The main issues they identified included the absence of disability-related demographic data in many datasets (limiting bias detection), the inadequacy of fairness metrics that assume static user traits, and the failure of systems to accommodate users with assistive technologies or non-normative interaction patterns. The paper demonstrated how AI systems produce disparate outcomes for users with disabilities like facial recognition systems inaccurately identifying wheelchair users, or job-matching algorithms penalizing non-standard educational timelines associated with disability. The authors proposed a set of actionable considerations for inclusive AI design, including integrating flexibility into AI models, embedding inclusive testing methodologies, and actively involving disabled people in the development pipeline. This work offers essential guidance for integrating multi-dimensional bias detection and mitigation practices into TEVV frameworks, ensuring that AI systems are verified and validated with attention to inclusive fairness and representational completeness.

Whittaker et al. (40) conducted a comprehensive analysis of AI systems' impact on disabled communities through policy review, case study analysis, and stakeholder interviews with disability rights organizations. Their investigation revealed that traditional privacy protection methods like k-anonymity become ineffective for small disability populations, where unique characteristics make individuals easily identifiable even in anonymized datasets. The study documented specific cases where AI systems created disproportionate surveillance risks for disabled users, including biometric authentication systems that required multiple attempts for users with motor disabilities, creating detailed behavioral profiles, and predictive policing algorithms that disproportionately targeted disabled individuals through behavioral pattern analysis. Their analysis identified three critical privacy vulnerabilities: insufficient anonymization for rare disabilities, excessive data collection requirements for accessibility features, and inadequate consent mechanisms for users with cognitive disabilities. This comprehensive privacy impact analysis provides essential ethical validation requirements for TEVV frameworks by establishing protocols for assessing and mitigating privacy risks specific to disabled populations in AI accessibility systems.

### 2.3 Accessibility metrics and evaluation criteria

Goggin et al. (1) conducted a comparative policy analysis of digital accessibility frameworks across Australia, United States, European Union, and United Kingdom, examining regulatory approaches, implementation strategies, and compliance mechanisms through document analysis and stakeholder interviews. Their investigation identified three emerging standardization trends: mandatory conformance testing requirements, establishment of certified evaluation bodies for third-party assessment, and transparency reporting obligations requiring public disclosure of accessibility compliance status. The study revealed significant variations in enforcement mechanisms, with some jurisdictions relying on complaint-based systems while others implemented proactive monitoring programs. Their analysis demonstrated that countries with stronger regulatory frameworks achieved higher accessibility compliance rates across government digital services. This comparative framework analysis provides essential standardization guidance for TEVV methodologies by establishing internationally recognized benchmarks and compliance requirements that must be integrated into comprehensive testing and validation protocols. Work on Accessible, Explainable, Artificial Intelligence (AXAI) reinforces the need for accessibility-aware metrics by emphasizing that evaluation of AI explanations must account for modality, cognitive effort, and usability for disabled users, not for their technical faithfulness to underlying models exclusively (11).

Kumar et al. (4) proposed a new benchmarking protocol for PDF accessibility, using large-scale automated and LLM-based approaches to demonstrate how harmonized, statistical metrics can standardize accessibility assessment across contexts. Paddison and Englefield (13) and Andreia et al. (14) demonstrated the synergy of expert-driven heuristics and concrete accessibility guidelines, offering evidence that blended evaluation models yield comprehensive outcomes for TEVV implementations.

Morris et al. (5) conducted a mixed-methods investigation into the accessibility and use of Twitter by people who are blind. The study combined an online survey of 132 blind Twitter users with large-scale quantitative analysis of user profiles and tweets, including comparisons to a matched group of sighted users. Survey questions explored motivations, experiences, and barriers associated with Twitter use, as well as specific challenges related to image-based content and profile customization. Their findings could be grouped into five areas: profile and content customization barriers (many cited accessibility challenges, lack of awareness, or the difficulty of confirming visual changes as reasons for retaining default images), increasing visual content and access gaps (despite the sharp rise in embedded imagery on Twitter and image-based tweets, none of the images included textual descriptions, making them inaccessible to screen reader users and limiting blind users' participation in image-rich interactions and trending content), distinctive usage patterns (blind Twitter users wrote longer bios, used a more advocacy-oriented set of hashtags, and were substantially less likely to retweet or post images compared to sighted users—their tweet volume per day, however, was statistically similar, underscoring high engagement despite accessibility challenges), privacy risks and predictive features (logistic regression showed that blind users could be identified with over 90% accuracy based on profile and usage patterns, raising concerns about inadvertent privacy and discrimination risks associated with disability status signals), and community-reported needs (the majority of blind users voiced strong interest in more consistent and meaningful image descriptions—difficulty accessing multimedia content drove some to unfollow accounts or use workarounds, reflecting ongoing social exclusion as Twitter's ecosystem became more visual in nature). These findings highlight the limitations of traditional usability assessment approaches, which may miss accessibility obstacles specific to evolving content formats and social interactions. The methodology—blending direct user feedback, behavioral data, and quantitative analyses—demonstrates an effective approach for TEVV protocols in AI accessibility, supporting the development of measurement frameworks sensitive to real-world usage barriers, representational differences, and evolving platform practices.

### 2.4 User-centered approaches

Kane et al. (12) and Sarsenbayeva et al. (6) showed, through survey and co-word analysis, the importance of involving diverse populations in all evaluation phases to surface hidden accessibility failures and iteratively refine AI systems (6, 12). Xiao et al. (15) further advocate for ability-diverse collaboration through systematic, long-term participatory methods (15). Correia et al. (16) confirmed, through comparative testing, that multi-modal interfaces (e.g., voice and touch controls) can mitigate barriers for motor-impaired users, reinforcing the need for user-driven multi-modal testing protocols in TEVV. Additional work on participatory and co-design approaches with disabled people further demonstrates how collaborative methods can surface context-specific accessibility barriers and inform AI design decisions across domains, including health, education, and digital services (27–31).

### 2.5 Ethical considerations

Korada et al. (17) present a detailed conceptual framework synthesizing artificial intelligence and established accessibility principles to guide the development of inclusive technologies for people with disabilities. The authors performed literature analysis and critical engagement with policy, technical, and social perspectives, to emphasize that AI deployments must be grounded in both universal design and rights-based models of disability. The framework articulates the need for adaptable and configurable AI systems—tools that prioritize flexibility to meet the individualized requirements of diverse users across learning, employment, and social domains. The operational guidance offered includes explicit recommendations for collaborative development processes, continuous post-deployment monitoring to detect and address emergent bias, systematic stakeholder engagement, and context-sensitive evaluation metrics that reflect the lived experiences and evolving needs of disabled users. This informs TEVV's operational practices for continuous stakeholder input and bias monitoring, echoing findings from Bobek et al. (18), who demonstrate the impact of user-centric explainability and transparency measures. These approaches establish foundational validation criteria for TEVV protocols: it ensures that ethical review, privacy, fairness, and participatory design are systematically prioritized in all stages of accessible AI system development and operation. Broader ethical and sociotechnical scholarship on AI, disability, and accessibility also highlights the need for continuous monitoring, cross-sector collaboration, and shared accountability mechanisms that extend beyond any single AI system to the wider media and information ecosystem (36–39).

### 2.6 Regulatory and standards-based foundations

Recent work on accessibility standards demonstrates the central role of formal guidelines in modern AI evaluation. Sharma et al. (19) conducted a large-scale analysis of web content from ophthalmology social media to systematically assess compliance with WCAG 2.1 guidelines using both automated tools and manual inspection. Their finding revealed widespread accessibility gaps, especially in contrast and alt text quality, despite broad awareness of these standards. This study's dual method evaluation process illustrates best practices for integrating regulatory metrics into TEVV and highlights the persistent need for human-in-the-loop validation when auditing AI for accessibility. The World Wide Web Consortium (W3C) provides foundational documentation for WCAG 2.0 and 2.1, which establish essential benchmarks for accessibility testing in all relevant digital environment.

ISO standards have recently informed robust evaluation criteria for usability. The ISO 9241-210 (2019) standard defines human-centered design processes and operationalizes usability constructs for interactive systems. It provides prescriptive guidance on user involvement, iterative testing, and context-specific evaluation, which directly supports the structure of TEVV, allowing for systematic incorporation of user feedback and iterative refinement throughout the AI lifecycle.

Nielsen's usability heuristics (Nielson 1994) continue to be influential in accessibility testing. Zhang and Walji (41) applied extended heuristic evaluation methods to clinical dashboard design and demonstrated how the use of expert-based heuristics contributed to improved interface accessibility for clinicians with varied expertise. These findings reinforce the importance of usability heuristics in the TEVV framework, both as a means of expert validation and as an actionable checklist for real-world evaluation. Recent analyses of evolving legal requirements and standards for digital and AI accessibility reinforce that future-facing TEVV frameworks must align not only with WCAG but also with emerging national and international regulations governing government and healthcare websites, AI-enabled services, and data governance (32–35).

### 2.7 TEVV, fairness frameworks, and inclusive AI

Standards-based research is now complemented by more specialized frameworks that target AI risk and fairness. The NIST AI Risk Management Framework (2022) offers a structured approach for identifying and mitigating risks in generative AI systems, including rigorous documentation and evaluation procedures. Its modularity and focus on risk profiles allow for tailored testing strategies that are essential for disability-inclusive TEVV.

Recent medical informatics research also addresses disability equity in AI. Umucu (42) propose an integrated evaluation framework that includes participatory design, iterative user feedback, and specific fairness metrics tailored to disability contexts. Their findings show that collaborative development processes can significantly reduce harmful bias and exclusion in AI-driven healthcare applications. These methodological innovations are directly relevant for structuring TEVV protocols and emphasize the need to move beyond surface-level compliance toward outcomes-based fairness validation.

### 2.8 Empirical insights from HCI and disability studies

Longitudinal and user-centered approaches have become vital in rigorous accessibility evaluation. Alonzo and Hasaan (20) reviewed 25 years of HCI research on digital reading for people with disabilities and documented how modality-specific barriers persist in mainstream digital environments. Their systematic review identifies electronic reaching formats and assistive technologies as key leverage points for accessibility improvements in AI. This historical breadth provides TEVV frameworks with validated methods for benchmarking progress and structuring long-term evaluation.

Aljedaani (44) analyzed how government websites' compliance with accessibility standards directly influenced user satisfaction and navigation rates. Their study confirmed that systematic adoption of accessibility regulations leads to measurable gains in usability for disabled users, reinforcing the imperative to incorporate international standards into all phases of TEVV. Current scoping reviews of AI and disability research by Umucu et al. (43) offer comprehensive harmful bias and usability evaluations identifying persistent disparities in accuracy, representation, and adaptivity for disabled populations. Their synthesis provides evidence-based priorities for continuous testing and verification, which strengthens the empirical foundations of the accessibility TEVV framework.

Umucu (42) reviewed collaborative work models for people of varying abilities and found that active co-design and longitudinal participation yield significantly better outcomes in AI system usability and satisfaction. Their systematic review provides methodological support for participant recruitment, mixed methods validation, and continuous evaluation in accessibility-focused TEVV.

## 3 Background and current practices

### 3.1 The need for accessibility in AI

Ensuring the accessibility of AI is not just a matter of compliance or good practice, but a fundamental necessity for creating an inclusive society [(1); WCAG 2.0; W3C]. AI technologies are being deployed in a variety of ways across all segments of society. These systems are being used in healthcare for diagnosis and treatment planning, in education for personalized learning, in employment for resume screening and candidate selection, and in public services for information access and decision-making. If these tools are not accessible to people with disabilities, it can lead to significant barriers in accessing essential services, employment opportunities, and information.

Inaccessible AI can perpetuate and even amplify existing harmful societal biases and inequalities (21). For example, if speech recognition systems struggle to understand diverse speech patterns, including those associated with certain disabilities, it could lead to exclusion from voice-controlled devices and services. Similarly, if computer vision algorithms are not trained on diverse datasets that include people with various disabilities, they may fail to accurately identify or assist these individuals in applications ranging from autonomous vehicles to security systems (21).

### 3.2 Accessibility features and universal benefits

Before expanding into the specifics of the proposed TEVV framework for AI accessibility, it is important to understand the broader context of accessibility features and their universal benefits. This section provides an overview of key accessibility features across different modalities and illustrates how these features, while essential for users with disabilities, often enhance the experience for all users. Examination of the wide-ranging benefits of accessibility improvements allows for a potentially deeper appreciation of the importance of integrating comprehensive accessibility testing into AI development processes. Table 1 outlines various accessibility features, their specific benefits for users with accessibility needs, and the extrapolated universal advantages they offer to all users. The patterns summarized in Table 1 are consistent with prior accessibility and universal design research that shows that features such as high contrast modes, captions, voice control, and adjustable pacing function as classic “curb cut” effects: they are essential for some users with disabilities, but also measurably improve usability, flexibility, satisfaction, and utility for the broader user population (1, 5, 11).

**Table 1:** Outlines various accessibility features their specific benefits for users with accessibility needs, and the universal advantages they offer to all users.

Modality	Accessibility feature	Benefits for accessibility needs	Universal benefits
Visual	High contrast modes	Improves readability for users with low vision	Enhances visibility in bright environments for all users
	Screen reader compatibility	Enables navigation for blind users	Supports hands-free operation and multitasking
	Adjustable text size	Aids users with visual impairments	Improves readability on various screen sizes
Auditory	Closed captions	Essential for deaf and hard of hearing users	Useful in noisy environments or when audio is unavailable
	Transcripts	Provides text alternative for audio content	Allows quick scanning of content and improves searchability
	Volume normalization	Helps users with hearing sensitivity	Enhances audio clarity in varying environments
Motor	Voice control	Crucial for users with limited mobility	Enables hands-free operation for convenience
	Keyboard navigation	Essential for users who can't use a mouse	Improves efficiency for power users
	Customizable input methods	Accommodates various physical limitations	Allows personalization for comfort and efficiency
Cognitive	Clear, simple language	Aids users with cognitive disabilities	Improves comprehension and reduces cognitive load for all
	Consistent layout	Helps users with memory or attention difficulties	Enhances usability and reduces learning curve
	Customizable pacing	Accommodates varying processing speeds	Allows users to consume content at their preferred pace

**Table 2:** Taxonomy of accessibility considerations in AI systems.

Category	Subcategory	Examples
Sensory	Visual, Auditory	Screen reader compatibility, color contrast, image descriptions, captioning, audio descriptions, volume control
Motor	Input Methods, Physical Interaction	Voice control, eye-tracking, switch access, Gesture recognition, touch sensitivity
Cognitive	Information Processing, Learning	Clear language, adjustable pace, memory aids, adaptive interfaces, customizable difficulty levels
Speech	Recognition, Output	Diverse accent handling, speech impediment accommodation, natural language generation, speech synthesis quality
Neurodiversity	Attention, Sensory Processing	Distraction reduction, focus assistance, Adjustable sensory input, overload prevention
Situational	Environmental, Temporary Impairments	Noise adaptation, lighting adjustment, one-handed operation, simplified interfaces

**Table 3:** Taxonomy of TEVV methods for AI accessibility.

Method	Focus	Techniques	Outcomes
Red Teaming	Vulnerability Discovery, Bias Uncovering	Adversarial testing, edge case simulation, scenario development, systematic probing	Identified accessibility exploits and weaknesses, revealed hidden biases against users with disabilities
Model Testing	Performance Evaluation, Bias Detection	Diverse dataset testing, accessibility-specific metrics, statistical analysis, fairness testing	Quantitative assessment of model accessibility, identified and quantified accessibility biases
Field Testing	Real-world Performance, Integration Assessment	Long-term deployment, environmental testing, assistive technology compatibility testing	Insights into real-world accessibility challenges, evaluation of system interoperability
Usability Testing	User Experience, Accessibility heuristics	Task-based scenarios, think-aloud protocols, expert evaluation, accessibility checklist application	Qualitative feedback on accessibility and usability, systematic identification of usability barriers

How Are Improvements in Accessibility Beneficial to Everyone?

Enhancing accessibility in AI systems and technologies can offer widespread benefits that extend far beyond the specific needs of users with disabilities. These improvements can lead to more versatile, user-friendly, and robust products that benefit all users.

Accessibility features frequently improve the overall usability of a system. For example, clear and simple language designed to aid users with cognitive disabilities also reduces cognitive load for all users to make interfaces more intuitive and easier to navigate. Many accessibility features prove valuable in various situations. Closed captions, originally designed for deaf users, benefit anyone watching video in noisy environments or when they need to keep the volume low. These universal benefits indicate that accessibility features should be treated as core quality attributes from a TEVV perspective, with explicit test conditions and metrics that capture both disability-specific and general usability benefits.

Features like voice control and keyboard navigation, which can benefit users with motor disabilities, offer all users more flexibility in how they interact with technology. Implementation of features like these has the potential to lead to increased efficiency and reduced physical strain. Likewise, designing for screen readers often results in better-structured content, which can potentially improve navigation and comprehension for all users. Another example is seen in how ease in navigation can enhance search engine optimization and overall information architecture.

Voice recognition technology is another example of a tool that was initially developed to aid users with disabilities. It has since evolved into widely used virtual assistants. Considering accessibility from the outset can cultivate a more inclusive design process, that leads to products that are more versatile and accommodating of diverse user needs and preferences.

### 3.3 Current TEVV practices

Traditional software testing methodologies, while foundational, have significant limitations when applied to AI systems-especially in the context of accessibility (7, 18). These established approaches typically emphasize metrics like accuracy and general system performance (10). These approaches often fall short in comprehensively addressing the nuanced and dynamic accessibility needs of people with disabilities.

### 3.4 Why traditional TEVV falls short for accessibility

Surface-Level Compliance: Standard TEVV practices tend to focus on pass/fail checks (e.g., whether alt text is present), but may overlook whether accessibility features are contextually appropriate or provide genuine utility for people with disabilities. Treating accessibility as a post-deployment add-on rather than an integral part of the AI development process, can lead to superficial implementations that don't truly address user needs.

Limited Contextual Understanding: Traditional and even some AI-driven tools struggle to interpret the real-world experiences of users with disabilities, such as navigating a website with a screen reader or interacting with assistive technologies.

Lack of Specialized Accessibility Metrics:

The lack of specialized accessibility metrics in many existing evaluation frameworks hinders the comprehensive assessment of AI systems' accessibility performance. It can be challenging to quantify and compare the accessibility features of different AI solutions while these gaps remain.

Insufficient Representation:

Insufficient representation of people with disabilities in test datasets and user studies leads to significant evaluation gaps. Underrepresentation can result in AI systems that fail to address the unique needs and challenges faced by diverse user groups.

Static Testing Approaches: Traditional testing methods may not capture the dynamic nature of AI systems and how they interact with users with disabilities over time. AI behaviors can evolve and adapt and that necessitates more flexible and ongoing evaluation methods.

### 3.5 Proposed framework for AI TEVV for accessibility

To address the limitations of current practices and ensure comprehensive accessibility evaluation of AI systems, the following framework has been proposed:

#### 3.5.1 Framework overview

The proposed AI TEVV for accessibility framework consists of four main components:

Red Teaming
Model Testing
Field Testing
Usability Testing

**Figure 1:**
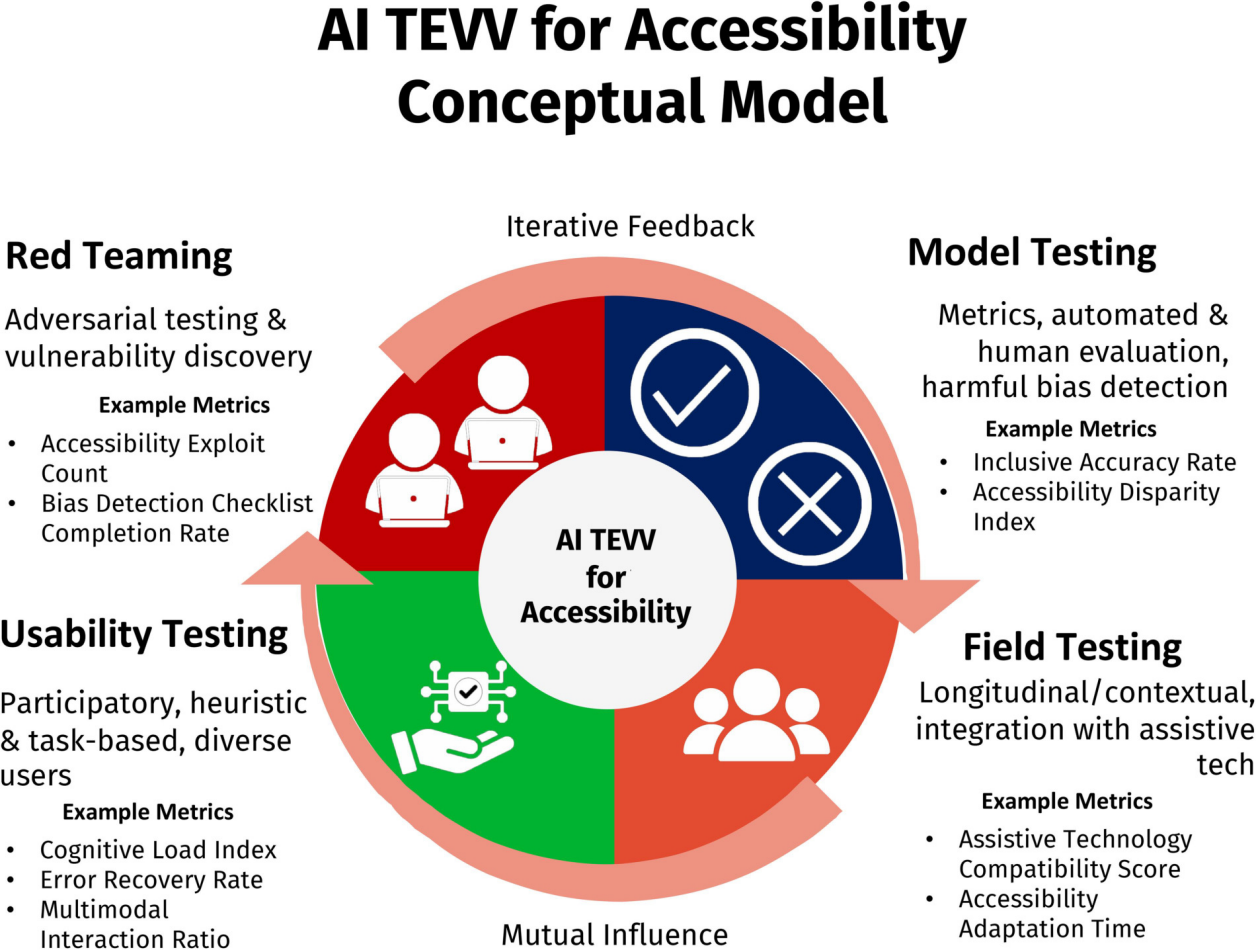
AI TEVV for accessibility conceptual model. Icons adapted from Adobe Stock: “Set of computer user icons with wi-fi symbols in various styles” by Coosh448, “green checkmark and red x cross icons in circles for yes no approval rejection decision isolated on white background” by Samaia, “Accesibility Icon Collection Glyph & Mixed Style” by Naba and “group glyph icon” by Sentya, licensed under Enhanced License.

At the core of the conceptual framework, visualized in Figure 1, is the recognition that testing, evaluation, verification, and validation are not discrete phases but overlapping, mutually informing activities. By explicitly integrating red teaming, model testing, field testing, and usability testing, and linking each to both specialized accessibility metrics and real-world user feedback, the TEVV model creates a continuous feedback loop. This enables the identification and remediation of harmful biases, usability barriers, and technical failures across diverse scenarios and populations. Ultimately, the model seeks to move accessibility from a compliance checkbox to a catalyst for innovation in AI system design and deployment.

The AI TEVV for accessibility framework is grounded in the normative and methodological tenets of universal design, AI ethics, and established technical standards. Universal design, as articulated in ISO/IEC 25010 and referenced in WCAG 2.2 and EN 301 549, requires that systems accommodate diverse users by default, rather than as an add-on consideration. The AITEVV for accessibility approach operationalizes this mandate by embedding accessibility and harmful bias mitigation within each phase of AI system development (ISO/IEC 25010; WCAG 2.2; EN 301 549). The framework also incorporates guidance from contemporary AI ethics, mandating participatory involvement of disabled stakeholders, transparency, and iterative fairness auditing to align with both European and international accessibility legislation and technical standards (9, 22). This explicit integration provides conceptual coherence to situate the AI TEVV for accessibility framework within the broader movement toward rights-based, ethically sound, and universally inclusive technology systems.

Taxonomies play an important role in structuring and standardizing the complex space of accessibility evaluation in artificial intelligence systems. By systematically categorizing the various types of user needs, interaction modalities, barriers, evaluation methods, and potential system vulnerabilities, the Taxonomy of Accessibility Considerations in AI Systems (Table 2) and the Taxonomy of TEVV Methods for AI Accessibility (Table 3) provide a foundational framework that guides both the design of testing protocols and the interpretation of findings. The goal of organizing accessibility challenges and verification activities into clearly defined categories is to help researchers and practitioners to apply rigorous, repeatable assessment strategies, compare outcomes across studies, and target improvements more precisely. The taxonomies, grounded in both empirical studies and established standards, outline the essential dimensions and methods addressed by the TEVV framework, and serve as a blueprint for comprehensive and iterative accessibility evaluation.

Each component plays a role in evaluating different aspects of AI accessibility. The framework is designed to be iterative, with insights from each stage informing improvements and further testing in others.

### 3.6 Red teaming for accessibility

Red teaming in the context of AI accessibility involves simulating adversarial scenarios to uncover potential vulnerabilities or biases that could disproportionately affect users with disabilities. The process helps to identify edge cases and unexpected behaviors that might not be captured in standard testing procedures.

Key Aspects of Red Teaming for Accessibility:

Diverse Adversarial Team: Assembling a diverse adversarial team is necessary for effective red teaming in accessibility (12). This team should include experts in accessibility, disability rights advocates, and individuals with various disabilities to provide a comprehensive perspective on potential vulnerabilities. 2. Proxy Scenario Development: Proxy scenario development involves creating realistic situations that challenge the AI system's ability to accommodate different disabilities and assistive technologies (NIST ARIA, Evaluation Plan, 2024). These scenarios should cover a wide range of potential use cases and edge cases. 3. Ethical Considerations: Ethical considerations are critically important in red teaming activities to ensure they don't cause harm or distress to participants. Careful planning and execution of tests, with proper safeguards in place are required in this domain. 4. Systematic Probing: Systematic probing methodically tests the system's responses to various inputs and interactions that simulate different disabilities and use cases. This structured approach helps uncover hidden biases or accessibility barriers. 5. Documentation and Analysis: Thorough documentation and analysis of findings are essential for identifying patterns of accessibility failures or biases and forms the basis for targeted improvements and further testing.

### 3.7 Model testing for accessibility

Model testing focuses on rigorously evaluating the AI model's performance across diverse user profiles, with a particular emphasis on accessibility considerations (16). This stage involves both quantitative and qualitative assessments of the model's outputs and behaviors.

Key Components of Model Testing for Accessibility:

1. Diverse Test Datasets: Developing and using diverse test datasets that represent a wide range of disabilities, assistive technologies, and user scenarios is necessary for comprehensive model testing to ensure the AI system is evaluated across a broad spectrum of potential users. 2. Accessibility-Specific Metrics: Implementing specialized metrics to measure the model's performance in accessibility-related tasks, such as accuracy in speech recognition for diverse speech patterns, provides quantitative insights into the system's accessibility performance.3. Harmful Bias Detection: Employing statistical techniques for the detection of harmful bias helps identify potential discriminatory behaviors against users with disabilities in the model's outputs. Subtle biases that might not be apparent through other testing methods can be revealed through these techniques. Table 4: Statistical Methods for Bias Detection describes statistical methods for accessibility bias detection. Table 5: Beneficial Analysis for Bias Detection illustrates analyses that are beneficial for bias detection in accessibility. 4. Robustness Testing: Robustness testing evaluates the model's performance under various conditions that simulate real-world challenges faced by users with disabilities. This includes testing with imperfect inputs or in challenging environmental conditions. 5. Explainability Analysis: Assessing the interpretability of the model's decision-making process for decisions that impact accessibility, is an essential component for building trust and ensuring the system's reasoning aligns with accessibility principles.

**Table 4:** Statistical methods for bias detection.

Method	Description	Key steps
Disparate Impact Analysis	Compares outcomes for users with and without disabilities	• Calculate selection rate for each group • Compute ratio of rates (bias indicator if < 0.8)
Demographic Parity	Assesses equality of positive outcome probability across groups	• Compare positive outcome proportions • Use chi-square tests for significance
Equal Opportunity Difference	Focuses on true positive rates across groups	• Calculate true positive rate for each group • Compare rates using z-tests or *t*-tests
Average Odds Difference	Combines equal opportunity and false positive rates	• Compute true and false positive rates for each group • Average differences for a single bias metric
Theil Index	Measures outcome entropy across groups	• Calculate entropy of model predictions for each group • Higher values indicate more unequal distribution
Propensity Score Matching	Isolates disability status effect on outcomes	• Match users based on relevant features • Compare outcomes between matched pairs

**Table 5:** Beneficial analyses for bias detection.

Analysis type	Key steps	Description
Intersectional Bias Analysis	• Use multivariate regression to assess interaction effects	Examines intersection of disability with other characteristics
Temporal Bias Analysis	• Conduct longitudinal studies • Use time series analysis for trends	Investigates bias changes over time
Feature Importance Analysis	• Apply SHAP values • Identify disproportionate influence of disability features	Determines features contributing to bias
Subgroup Fairness Analysis	• Stratify data by disability type/severity • Compare performance metrics across subgroups	Assesses performance across disability types/severities
Counterfactual Analysis	• Create synthetic data by altering disability features • Analyze prediction changes	Generates “what-if” scenarios
Bias Amplification Analysis	• Compare outcome distributions in training data vs. predictions • Quantify bias increase from data to output	Investigates model amplification of training data biases

The statistical methods in Table 4 draw on widely used group fairness metrics in machine learning, including disparate impact, demographic parity, and equal opportunity, which have been extensively discussed and compared in the fairness metrics literature (23, 24). Theil index based disparity measures and propensity score matching have similarly been used to quantify outcome inequality and isolate the effects of protected attributes in applied fairness studies in health and clinical AI contexts particularly (24).

The analysis types summarized in Table 5 are consistent with emerging best practices in AI fairness assessment. Intersectional and subgroup fairness analyses have been emphasized as necessary to reveal compounded harms at the intersection of multiple protected attributes (25). Temporal bias analysis addresses concerns that fairness properties can drift over time in deployed systems (24). Counterfactual and causal-inference-based approaches have been used to test whether predictions remain stable when protected attributes are hypothetically altered. This helps to detect and reason about discriminatory decision boundaries (25). Bias amplifications analysis comparing disparities in training data vs. model outputs, has been highlighted as essential for understanding how models may exacerbate existing inequities encoded in source data (24).

### 3.8 Key metrics for AI accessibility

Accessibility metrics are necessary for quantifying an AI system's performance in accommodating users with diverse abilities. These metrics were created to provide objective measures to assess improvements, compare different systems, and ensure that accessibility is consistently evaluated throughout the development process. This emphasis on measurable accessibility outcomes is consistent with recent AXAI scholarship, which argues that explanation quality should be assessed in terms of how accessible, understandable, and cognitively manageable explanations are for users with disabilities, not just for their correctness (11).

The proposed key metrics for AI accessibility have been introduced and outlined below. Table 6 describes the metrics and their proposed target values.

Inclusive Accuracy Rate (IAR): This metric measures the AI's accuracy across diverse user groups, including those with disabilities. IAR=(Correct predictions for users with disabilities/Total predictions for users with disabilities) * 100
Accessibility Disparity Index (ADI): This index quantifies the difference in performance between users with and without disabilities. ADI=(Accuracy for users without disabilities—Accuracy for users with disabilities)/Accuracy for users without disabilities
Assistive Technology Compatibility Score (ATCS): This score evaluates how well the AI system integrates with common assistive technologies. ATCS=(Number of successfully integrated assistive technologies/Total number of tested assistive technologies) * 100
Cognitive Load Index (CLI): This metric assesses the cognitive effort required to interact with the AI system, which is particularly important for users with cognitive disabilities. CLI=Weighted sum of factors like task completion time, error rate, and user-reported difficulty
Multimodal Interaction Ratio (MIR): This ratio measures the AI's ability to provide and process information through multiple modalities. MIR=Number of supported interaction modalities/Total number of possible interaction modalities
Accessibility Adaptation Time (AAT): This metric measures how quickly the AI system adapts to individual user needs and preferences. AAT=Time taken for the AI to reach optimal performance for a user with specific accessibility needs
Error Recovery Rate (ERR): This rate assesses how effectively users can recover from errors when interacting with the AI system. ERR=(Number of successfully recovered errors/Total number of errors) * 100

**Table 6:** Calculating the key metrics of accessibility.

Metric	Formula	Purpose	Target value
Inclusive Accuracy Rate (IAR)	(Correct predictions for users with disabilities/Total predictions) * 100	Ensure AI performs well for users with disabilities	≥ 95%
Accessibility Disparity Index (ADI)	(Accuracy for users without disabilities—Accuracy for users with disabilities)/Accuracy for users without disabilities	Measure performance gap between user groups	≤ 0.05
Assistive Technology Compatibility Score (ATCS)	(Successfully integrated ATs/Total tested ATs) * 100	Evaluate integration with assistive technologies	≥ 90%
Cognitive Load Index (CLI)	Weighted sum of task time, error rate, and user-reported difficulty	Assess ease of use for cognitive accessibility	≤ 3 (on a 1-10 scale)
Multimodal Interaction Ratio (MIR)	Supported interaction modalities/Total possible modalities	Measure flexibility in interaction methods	≥ 0.8
Accessibility Adaptation Time (AAT)	Time to reach optimal performance for users with specific needs	Evaluate AI's adaptability to individual requirements	≤ 5 interactions
Error Recovery Rate (ERR)	(Successfully recovered errors/Total errors) * 100	Assess system's ability to help users recover from mistakes	≥ 90%

The quantitative metrics used in the TEVV for accessibility framework were derived through a mixed approach: some indices adapt established measures from the human–computer interaction (HCI) and accessibility standards literature, while others are newly defined to address gaps in current evaluation practices.

Metrics such as error recovery rate, task success rate, and cognitive load index are adapted from widely used HCI usability measures and are in alignment with international standards such as ISO 9241-210 and the systems/software quality models in ISO/IEC 25010. Additional accessibility-driven measures such as the Inclusive Accuracy Rate (IAR), Accessibility Disparity Index (ADI), and Assistive Technology Compatibility Score (ATCS) were developed based on recommendations and operational definitions provided in sources like WCAG 2.2, EN 301 549, and meta-analytic reviews (1, 2, 4, 7). When devising new indices (e.g., IAR, ADI), formulas were constructed to reflect proportionality and subgroup fairness as outlined in recent accessibility and AI fairness research (9).

Threshold values for these indices were established in two ways:

For standard usability/accessibility metrics (e.g., task completion > 80%, error rate < 5%), target values were adopted from benchmarks commonly reported in HCI and accessibility evaluation studies and cross-referenced with threshold guidance in international standards (ISO/IEC 25010; WCAG 2.2; EN 301 549).
For newly defined or adapted metrics (e.g., ADI, ATCS), thresholds were initially set to match prevailing legal/regulatory cutoffs for accessibility conformance (e.g., WCAG AA, EN 301 549), and further justified through calibration using published case studies and empirical findings (5, 10).

All metrics and thresholds were reviewed for interpretability, relevance, and feasibility with reference to meta-analytic work and cross-case validation in the literature (2, 8). The resulting suite of indices allows for both standardized and context-sensitive evaluation of AI accessibility to enable benchmarking, iterative improvement, and meaningful comparison across different system implementations and user groups.

These metrics provide a comprehensive framework for evaluating AI accessibility. They cover various aspects of accessibility, from overall performance and fairness to specific considerations like assistive technology compatibility and cognitive load. By consistently measuring and optimizing these metrics, developers can create AI systems that are more inclusive and effective for users with diverse abilities.

Incorporating these metrics into the AI development process helps ensure that accessibility is not an afterthought but a fundamental consideration throughout. They provide concrete goals for improvement and allow for objective comparison between different AI systems or versions. These metrics can also help identify specific areas where an AI system may be falling short in terms of accessibility to assist in guiding targeted improvements and resource allocation.

It's important to note that these metrics should be used in conjunction with qualitative feedback and real-world testing to provide a holistic view of an AI system's accessibility. The target values provided are suggestions and may need to be adjusted based on the specific context and requirements of each AI application.

### 3.9 Field testing

Field testing involves assessing the AI system's real-world usability and accessibility in authentic contexts. This stage provides valuable insights into how the system performs outside of controlled environments.

Key Elements of Field Testing for Accessibility:

1. Diverse Testing Environments: Conducting tests in various real-world settings relevant to the AI system's intended use provides valuable insights into its performance across different environments. Examples might include homes, workplaces, and public spaces. 2. Long-term Evaluation: Long-term evaluation assesses the system's performance and accessibility over extended periods to capture evolving user needs and system behaviors (12). A longitudinal approach reveals how the AI adapts and performs over time. 3. Integration Testing: Integration testing evaluates how the AI system interacts with various assistive technologies and accessibility features of different devices to ensure compatibility and seamless operation with the tools many users rely on. 4. Contextual Factors: Considering environmental factors such as lighting, noise, and connectivity helps identify how these variables impact accessibility. This contextual understanding is needed to make certain the AI functions effectively in diverse real-world conditions. 5. User Feedback Collection: Gathering qualitative feedback from users with disabilities about their experiences with the AI system in real-world contexts provides invaluable insights that may not be captured through quantitative metrics alone.

### 3.10 Usability testing for accessibility

Usability testing focuses on conducting in-depth evaluations with users who have various disabilities to assess the system’s effectiveness, efficiency, and satisfaction from an accessibility perspective.

Key Components of Usability Testing for Accessibility:

1. Diverse Participant Recruitment: Diverse participant recruitment involves including users with a wide range of disabilities, assistive technology preferences, and skill levels to ensure the usability testing captures a comprehensive range of user experiences. 2. Task-Based Scenarios: Developing realistic tasks that reflect use cases for the AI system, tailored to different disability profiles, helps assess the system's effectiveness in real-world scenarios. These tasks should cover the full spectrum of the AI's intended functionality. 3. Mixed-Methods Approach: A mixed-methods approach combines quantitative metrics like task completion rates and error rates with qualitative feedback from think-aloud protocols and interviews for a holistic view of the system's usability. 4. Accessibility Heuristics: Applying specialized accessibility heuristics to evaluate the AI system's interface and interactions helps identify specific usability issues related to accessibility. These heuristics are designed to uncover common barriers faced by users with disabilities. 5. Iterative Testing: Conducting multiple rounds of usability testing throughout the development process allows for tracking improvements and identifying persistent issues. This iterative approach ensures continuous refinement of the AI system's accessibility features.

### 3.11 AI TEVV for accessibility notes

The AI TEVV for Accessibility Notes provide a structured method for documenting the accessibility testing process throughout the development lifecycle of AI systems. This format is designed to ensure comprehensive coverage of all aspects of the TEVV framework while maintaining consistency and clarity in documentation. The template is included below:

Template Structure

Project Name:

Date:

Tester Name:

Red Teaming (RT)

**Table:** 

Adversarial Team Composition:	
Scenarios Tested:	
Key Findings:	
Ethical Considerations:	

Model Testing (MT)

**Table:** 

Test Datasets Used:	
Accessibility-Specific Metrics:	
Bias Detection Results:	
Robustness Test Outcomes:	
Explainability Analysis:	

Field Testing (FT)

**Table:** 

Testing Environments:	
Duration of Evaluation:	
Integration Test Results:	
Contextual Factors Observed:	
User Feedback Summary:	

Usability Testing (UT)

**Table:** 

Participant Demographics:	
Tasks Evaluated:	
Quantitative Metrics:	
Qualitative Feedback:	
Accessibility Heuristics Applied:	

Assessment (A)

**Table:** 

Current Accessibility Status:	
Key Issues Identified:	
Root Cause Analysis:	
Comparison to Accessibility Goals:	

Plan (P)

**Table:** 

Immediate Actions:	
Long-term Strategies:	
Monitoring and Maintenance Plan:	
Next Testing Iteration:	

Guidelines for use

Complete all sections for each testing iteration.
Be specific and detailed in your observations and findings.
Use objective language and provide concrete examples where possible.
Update the notes regularly throughout the development process.
Ensure all team members have access to and understand how to use the notes.
Use the Assessment and Plan sections to drive continuous improvement.

Example of Filled-Out Notes

Project Name: VoiceAssist AI

Date: August 15, 2024

Tester Name: Alex Johnson

Red Teaming (RT)

**Table:** 

Adversarial Team Composition:	Speech therapist, deaf individual, AAC expert
Scenarios Tested:	Understanding diverse speech patterns, non-verbal commands
Key Findings:	System struggled with certain speech impediments and accents, limited AAC compatibility
Ethical Considerations:	Ensured testing didn't cause distress to participants

Model Testing (MT)

**Table:** 

Test Datasets Used:	Diverse speech recordings including various disorders and accents
Accessibility-Specific Metrics:	Speech recognition accuracy for impaired speech
Bias Detection Results:	Lower accuracy for certain speech impairments
Explainability Analysis:	Difficulty interpreting model's speech recognition decisions

Field Testing (FT)

**Table:** 

Testing Environments:	10 homes of users with various disabilities
Duration of Evaluation:	3 months
Integration Test Results:	Issues with some popular AAC devices
Contextual Factors Observed:	Background noise significantly impacted performance
User Feedback Summary:	Frustration with limited visual feedback options

Usability Testing (UT)

**Table:** 

Participant Demographics:	15 users with diverse disabilities (deaf, hard of hearing, speech disorders, motor impairments)
Tasks Evaluated:	System setup, common commands, error recovery
Quantitative Metrics:	Task completion rates, time on task, error rates
Qualitative Feedback:	Difficulties in error recovery for non-verbal users
Accessibility Heuristics Applied:	Applied: WCAG 2.2, Inclusive Design Principles

Assessment (A)

**Table:** 

Current Accessibility Status:	Significant improvements needed in speech recognition and AAC compatibility
Key Issues Identified:	Poor performance with impaired speech, limited visual feedback, difficult error recovery
Root Cause Analysis:	Insufficient diverse training data, lack of multimodal interaction options
Comparison to Accessibility Goals:	Falls short in speech recognition accuracy and AAC integration targets

Plan (P)

**Table:** 

Immediate Actions:	Enhance speech recognition model with more diverse data, improve AAC device integration
Long-term Strategies:	Develop visual interface, implement adaptive learning for individual users
Monitoring and Maintenance Plan:	Monthly performance reviews, quarterly user feedback sessions
Next Testing Iteration:	Scheduled for November 15, 2024, focus on speech recognition improvements and new visual interface

Teams using this template can ensure all critical aspects of accessibility testing are consistently documented; easily track progress and improvements over multiple iterations; facilitate clear communication among team members and stakeholders; and maintain a comprehensive record of accessibility considerations throughout the AI development process.

To implement the notes in practice, create a digital version of this template (e.g., in a shared document or specialized software); integrate it into existing development workflows and documentation processes; train team members on how to use and update the template throughout the development lifecycle; and regularly review and discuss the notes as a team to inform decision-making and prioritize accessibility improvements.

### 3.12 Framework integration

To maximize the effectiveness of the TEVV process, these components should be integrated and inform each other iteratively. For example, insights from red teaming can guide the development of more robust model testing scenarios to ensure that the AI system is evaluated against a comprehensive range of potential accessibility challenges. Field testing observations can inform the design of more realistic usability testing tasks to bridge the gap between controlled testing environments and real-world usage scenarios. Usability testing feedback can help prioritize areas for focused red teaming and model improvements to make certain that development efforts are directed towards the most impactful accessibility enhancements. Systematic application of this framework throughout the AI development lifecycle can support organizations in creating more accessible, inclusive, and effective AI systems.

#### 3.12.1 Comparison with current methods

Table 7: Comparison with Current Methods illustrates the advantages of the proposed framework and compares it with current TEVV practices.

**Table 7:** Comparison with current methods.

Aspect	Current methods	Proposed framework
Focus	General performance and fairness	Specific accessibility considerations
User Representation	Limited diversity in test data	Comprehensive inclusion of users with disabilities
Testing Approach	Often static and controlled	Dynamic, including real-world scenarios
Metrics	Generic accuracy and bias metrics	Specialized accessibility metrics
Adversarial Testing	General security focus	Accessibility-specific red teaming
User Involvement	Limited, often late in development	Extensive, throughout the development process
Contextual Factors	Often overlooked	Explicitly considered in field testing
Iterative Process	Variable	Integral to the framework
Explainability	General focus	Accessibility-specific interpretability

The proposed framework addresses many limitations of current methods by explicitly focusing on accessibility as a core consideration throughout the TEVV process; incorporating diverse perspectives and experiences of users with disabilities, which can lead to more inclusive and representative testing scenarios; combining controlled testing with real-world evaluation to provide a comprehensive assessment of the AI system’s accessibility performance; developing and applying specialized metrics for accessibility performance to allow for more precise and relevant evaluation of AI systems in terms of their usability for diverse users; and emphasizing the importance of context and long-term usability that recognizes that accessibility needs can change over time and in different environments.

### 3.13 Example case studies

To demonstrate utility and sufficiency, the TEVV for accessibility framework is illustrated through two applied scenarios drawn and synthesized from published studies and reports. Each scenario operationalizes the proposed methodology by mapping its phases, metrics, and outcomes to documented user experiences, as found in the inclusive design and accessibility evaluation literature (12, 16). These cases are not original empirical studies but serve to clarify how the framework would be implemented in practice and benchmarked against existing results. Comparative assessment tables highlight where TEVV for accessibility addresses limitations and evidence gaps documented in previous frameworks (7, 9, 10).

Example Case Study 1: Voice-Controlled Smart Home Assistant

Background: A technology company developed an AI-powered voice-controlled smart home assistant designed to help users manage various aspects of their home environment, including lighting, temperature, security, and entertainment systems.

Application of the Framework:

Red Teaming:

A team including speech therapists, deaf individuals, and experts in augmentative and alternative communication (AAC) devices was assembled.
They developed scenarios to test the system's ability to understand diverse speech patterns, respond to non-verbal commands, and integrate with various assistive technologies.
The red team uncovered that the system struggled with certain speech impediments and had limited compatibility with popular AAC devices.

Model Testing:

Diverse test datasets were created, including recordings of speech from individuals with various speech disorders, accents, and age groups.
Specialized metrics were developed to measure the system's accuracy in understanding and responding to diverse speech inputs.
Harmful bias detection revealed that the model had lower accuracy rates for users with certain types of speech impairments.

Field Testing:

The system was deployed in homes of users with various disabilities for a three-month period.
Researchers observed how environmental factors like background noise and room acoustics affected the system's performance for different users.
Long-term testing revealed that the system's performance degraded over time for some users as it failed to adapt to subtle changes in their speech patterns.

Usability Testing:

A diverse group of participants, including individuals who are deaf, hard of hearing, have speech disorders, and motor impairments, were recruited.
Tasks included setting up the system, performing common commands, and troubleshooting issues.
Think-aloud protocols and post-test interviews revealed frustrations with the system's limited visual feedback options and difficulties in error recovery for non-verbal users.

Outcomes: Based on the results of the AI TEVV for accessibility process, the development team obtained information that informed several improvements:

Improved the speech recognition model with more diverse training data (21).
Implemented better integration with popular AAC devices.
Developed a visual interface to complement voice controls.
Improved error handling and recovery mechanisms.
Implemented a learning algorithm to adapt to individual users' speech patterns over time.

These changes would significantly improve the system's accessibility and usability for a wider range of users.

Example Case Study 2**:** AI-Powered Job Application Screening System

Background: A large corporation implemented an AI system to screen job applications and identify promising candidates for human review. The system analyzed resumes, cover letters, and online assessments to rank applicants.

Application of the Framework:

Red Teaming:

The red team included disability rights lawyers, career counselors specializing in placing candidates with disabilities, and individuals with various disabilities who had experience with job searching.
They created scenarios to test how the system handled applications from candidates with disability-related employment gaps, alternative qualifications, and those requiring accommodations.
The team uncovered that the system was inadvertently penalizing candidates with non-traditional career paths often associated with certain disabilities.

Model Testing:

Test datasets were created that included a diverse range of resumes and application materials from candidates with various disabilities.
Metrics were developed to measure the system's fairness in ranking candidates with and without disabilities who had equivalent qualifications.
Harmful bias detection revealed that the model was less likely to highly rank candidates who disclosed certain disabilities or who had used disability-specific terms in their applications (9).

Field Testing:

The system was deployed in a controlled manner for actual job openings across several departments.
Researchers compared the AI system's rankings with those of human recruiters who were trained in inclusive hiring practices.
Long-term tracking of hired candidates' performance revealed no significant differences between those highly ranked by the AI system and those who had been potentially undervalued but hired through human intervention (12).

Usability Testing:

A diverse group of job seekers with various disabilities were recruited to go through a simulated application process using the AI system.
Tasks included uploading resumes, completing online assessments, and navigating the application portal.
Usability testing revealed significant barriers for users with visual impairments in completing certain types of online assessments, and difficulties for users with cognitive disabilities in understanding complex application instructions.

Outcomes: The results of the AI TEVV for accessibility process led to several important changes in the AI screening system:

Retraining of the model with more diverse datasets and adjusted weighting of factors to reduce harmful bias against non-traditional career paths.
Implementation of features to allow candidates to provide context for employment gaps or alternative qualifications.
Redesign of online assessments to be more accessible and offer multiple formats for demonstrating skills (17).
Improvement of the application portal's accessibility features, including better screen reader compatibility and simplified instructions.
Introduction of a human-in-the-loop process for reviewing applications flagged as potentially undervalued by the AI system due to disability-related factors.

These modifications could result in a more equitable screening process, increased diversity in the candidate pool, and improved overall satisfaction with the application experience for all users.

## 4 Discussion

The example case studies demonstrate the effectiveness of the proposed AI TEVV for accessibility framework for accessibility in uncovering and addressing critical issues that might have been overlooked using traditional testing methods. Systematic application of red teaming, model testing, field testing, and usability testing with a focus on accessibility can help organizations develop AI systems that are truly inclusive and effective for diverse user populations.

While the AI TEVV for accessibility framework is designed for comprehensive, lifecycle-wide evaluation, its modular structure enables practical adaptation for small and medium-sized organizations (SMEs). Each TEVV component, such as red teaming or field testing—can be implemented incrementally depending on available resources, with open-source toolkits and template guides provided for basic evaluations. Table 8 summarizes recommended minimal and optimal practice sets for organizations of varying sizes, emphasizing methods compatible with limited budgets and staff. Partnerships with disability organizations, use of synthetic or literature-sourced case studies, and phased metric adoption further facilitate scalable implementation (6, 26).

**Table 8:** AI TEVV for accessibility framework adaptation for varying organization sizes.

Organization size	Minimal required practices	Scalable enhancements
Small	Automated accessibility checkers; heuristic review; user feedback from community orgs	Add contextual field tests; partner with local advocacy groups
Medium	All small org. practices+participatory usability tests, red teaming with internal/external volunteers	Full metrics suite; cross-department training
Large	All above+longitudinal evaluation, expert/facilitated red teaming, in-depth metrics and integration testing	Continuous evaluation cycle, external audit, benchmark reporting

Existing frameworks [including those by (1, 10)] mainly treat accessibility as a compliance feature or *post hoc* evaluation. In contrast, the proposed TEVV framework centers accessibility as an iterative design and engineering concern. Evidence for this gap is supplied through examples in Table 9 below.

**Table 9:** Contrasting examples of existing frameworks with AI TEVV for accessibility.

Feature/approach	Existing frameworks (1, 10)	Proposed AI TEVV for accessibility framework	Supporting evidence/citations
Accessibility focus	Compliance, *post hoc* or checklist-based evaluation	Continuous, iterative, and central in design/development	(2, 7, 8)
Integration across lifecycle	Most apply accessibility after main development or as secondary consideration	Accessibility embedded as an ongoing, dynamic process	(1, 4)
Evaluation methods	Emphasis on static audits, technical guidelines, or automated tools	Multi-modal: red teaming, usability, model, and field testing	(9, 10)
User participation	Limited or delayed, typically in summative/usability testing	Diverse user involvement in all phases (design, metrics, validation)	(5, 12)
Bias, fairness, and context sensitivity	Under-addressed or unreported in typical frameworks	Explicitly targets bias detection, contextual factors, and fairness	(6–8)
Adaptability and real-world readiness	Often assumes static requirements and environments	Iterative, designed for adaptive deployment in evolving contexts	(2, 3)
Validation benchmarks	Rarely benchmarked with international standards or continuous improvement	Benchmarked against ISO, NIST, and WCAG, with clear improvement cycles	(4); National Institute of Standards and Technology, 2022

### 4.1 Key insights

Intersectionality of Accessibility Concerns

The framework reveals how accessibility issues often intersect with other forms of harmful bias and usability concerns, and highlighs the need for a holistic approach to AI development that considers multiple dimensions of diversity and inclusion.

Importance of Diverse Perspectives

Involving individuals with various disabilities throughout the TEVV process uncovers issues that might not be apparent to developers or traditional testers, underscoring the importance of diverse perspectives in AI development.

Dynamic Nature of Accessibility

The field-testing component highlights how accessibility needs can change over time and in different contexts and emphasizes the need for AI systems to be adaptable and continuously evaluated.

Complementary Nature of Framework Components

Each component of the framework provides unique insights that inform and enhance the others, which can lead to more comprehensive improvements than any single testing method alone.

Broader Benefits of Accessibility Focus

Many of the improvements made to enhance accessibility also benefit users without disabilities, which can lead to overall better user experiences and more robust AI systems.

### 4.2 Challenges in implementation

While the proposed framework offers significant benefits, several challenges may arise in its implementation.

Resource Intensity: The comprehensive accessibility testing required by this framework demands significant time, expertise, and financial resources, which may be challenging for smaller organizations or projects with limited budgets. Recruitment of Diverse Participants: Finding and engaging a truly diverse group of individuals with disabilities for testing can be challenging, especially for specialized AI applications that may require participants with specific experiences or skills. Balancing Competing Priorities: Organizations may struggle to balance accessibility improvements with other development priorities, especially when facing time or budget constraints. This requires careful project management and a strong commitment to accessibility. Evolving Accessibility Standards: As technology and societal understanding of disability evolve, accessibility standards and best practices may change, which will necessitate ongoing updates to the TEVV process to remain current and effective. Ethical Considerations: Involving individuals with disabilities in testing, especially in red teaming exercises, requires careful ethical consideration to ensure participants are not exploited or exposed to undue stress (40). This requires robust ethical guidelines and oversight.

Addressing intersectionality in the TEVV framework involves collecting and analyzing data that reflects multiple, interacting demographic and experience variables, such as disability status, gender, linguistic background, socioeconomic class, and age. During model and field testing, evaluation datasets should be intentionally stratified (or oversampled) for intersectional subgroups, and statistical techniques like multivariate regression and subgroup fairness metrics (e.g., conditional parity, intersectional bias indices) should be applied to analyze interactions (8, 9). Example: Fairness analyses examining “accuracy for non-native speakers with vision impairment” vs. ‘accuracy for other users can surface compound disparities overlooked by single-category tests. Researchers should report intersectional statistics, monitor adverse impact ratios for subgroups, and adjust red teaming and usability protocols to ensure scenarios reflecting these cross-cutting identities are developed and tested (2, 7). Additional granularity can be achieved via participatory feedback and qualitative think-aloud protocols targeting intersectional user experiences. Table 10 provides examples of these approaches.

**Table 10:** Intersectional measurement examples.

Variable 1	Variable 2	Example interaction	Measurement approach
Disability	Gender	Differential effect of accessibility features	Cross-tabulation; subgroup regression
Disability	Linguistic status	Voice UI bias against non-native speakers with impairment	Disaggregated accuracy by subgroup
Disability	Socioeconomic class	Access to assistive tech/modality preference	Survey/qualitative stratified analysis

### 4.3 Future directions

The proposed framework opens up several avenues for future research and development.

1. Automated Accessibility Testing: Developing AI-powered tools that can automatically detect certain types of accessibility issues in AI systems could complement human-led testing efforts, and potentially increase efficiency and coverage of accessibility evaluations (3). 2. Standardized Accessibility Metrics: Creating and/or adopting industry-wide standardized metrics for evaluating AI accessibility would allow for better comparison and benchmarking of different systems and promote transparency while driving improvements across the field. 3. Adaptive AI for Accessibility: Exploring how AI systems can dynamically adapt to individual users' accessibility needs over time, informed by insights from long-term field testing, could lead to more personalized and effective accessibility solutions. 4. Intersectional Analysis: Developing more sophisticated methods for analyzing how accessibility interacts with other dimensions of diversity and inclusion in AI systems could provide a more nuanced understanding of the complex dynamics between various user characteristics. 5. Regulatory Frameworks: Investigating how this TEVV framework could inform the development of regulatory standards for AI accessibility could help establish clear guidelines and expectations for accessible AI development across industries. 6. Educational Integration: Incorporating accessibility-focused TEVV methodologies into AI and computer science curricula to cultivate a new generation of accessibility-minded AI developers creates opportunities for innovation with methods for enhancing accessibility that are “baked in” and not “bolted on.”

While the proposed framework synthesizes the strongest elements of AI accessibility evaluation research, its narrative and validation are limited to thorough theoretical modeling and adaptation from the literature. Absence of new empirical data collection means real-world generalizability of implementation and user experience remains to be tested in future work (6, 18). The framework's novelty rests in its explicit, multi-phase integration and the demonstration, by comparative analysis, that prior models primarily treat accessibility as an adjunct or afterthought, rather than as a central, iterative driver of system design (1, 2, 4).

## 5 Conclusion

The proposed framework for AI Testing, Evaluation, Verification, and Validation (TEVV) focused on accessibility represents a significant step forward in ensuring that AI systems are truly inclusive and effective for all users. This approach integrates specialized methods for red teaming, model testing, field testing, and usability testing, to provide a comprehensive strategy for identifying and addressing accessibility barriers in AI technologies.

The example case studies demonstrate the framework's effectiveness in uncovering critical issues that might have been missed by traditional testing methods. They also highlight how addressing accessibility concerns can lead to overall improvements in AI system performance and user satisfaction for all users, not just those with disabilities.

As AI continues to play an increasingly central role in our society, the importance of accessibility in these systems cannot be overstated. Inaccessible AI risks perpetuating and even amplifying existing inequalities, while accessible AI has the potential to dramatically improve the lives of people with disabilities and create more inclusive digital and physical environments for everyone.

The challenges in implementing this framework are not insignificant, and require substantial resources, expertise, and commitment from organizations developing AI systems. However, the potential benefits—both ethical and practical—make this investment worthwhile. By adopting this accessibility-focused AI TEVV framework, organizations can:

Develop more robust and versatile AI systems.
Expand their potential user base and market reach.
Mitigate legal and reputational risks associated with inaccessible technologies.
Contribute to a more inclusive and equitable technological landscape.

As we move forward, continued research and collaboration between AI developers, accessibility experts, and individuals with disabilities will be essential in refining and expanding this framework. Prioritizing accessibility in AI development and rigorously testing for it, provides a path towards a future where AI technologies empower and include all members of society, regardless of ability.

The journey towards fully accessible AI is ongoing, but with frameworks like the one proposed here, the tools to make significant strides in the right direction may be within reach. It is incumbent upon all stakeholders in the AI ecosystem—from developers and researchers to policymakers and end-users—to advocate for and implement robust accessibility testing in AI development to ensure that the transformative potential of AI is realized equitably for all.

## Data Availability

The original contributions presented in the study are included in the article/Supplementary Material, further inquiries can be directed to the corresponding author/s.
